# Prevalence and Trend of Major Transfusion-Transmissible Infections among Blood Donors in Western China, 2005 through 2010

**DOI:** 10.1371/journal.pone.0094528

**Published:** 2014-04-08

**Authors:** Yan Song, Ying Bian, Max Petzold, Carolina Oi Lam Ung

**Affiliations:** 1 Institute of Chinese Medical Sciences, University of Macau, Taipa, Macau, China; 2 Center for Applied Biostatistics, Sahlgrenska Academy, University of Gothenburg, Gothenburg, Sweden; National Institute of Allergy and Infectious Diseases, United States of America

## Abstract

**Background:**

The prevalence of transfusion-transmissible infections (TTIs) in blood donations is important for evaluating blood safety and potential risks to the population. This study investigated the prevalence of TTIs among blood donors in Western China and suggested measures for policy-makers.

**Methods:**

The screening results of 66,311 donations between 2005 and 2010 from a central blood center in Western China were analyzed. The prevalence of hepatitis B virus (HBV), hepatitis C virus (HCV), human immunodeficiency virus (HIV), and syphilis infections were expressed in percentages for the entire study group as well as groups by demographic characteristics and donation frequency, with differences analyzed using Fisher's exact or Chi-square test. Logistic regression was performed to identify the influencing factors of the detected results.

**Results:**

1,769 (2.67%, 95% CI 2.55–2.79%) of the donated blood had serological evidence of infection with at least one pathogen and 44 (0.07%, 95% CI 0.05–0.09%) showed evidence of multiple infections. The seroprevalence of HBV, HCV, HIV, and syphilis infections was 0.87% (95% CI 0.80–0.94%), 0.86% (95% CI 0.79–0.93%), 0.31% (95% CI 0.26–0.35%), and 0.70% (95% CI 0.64–0.76%) respectively. Trend analysis for the prevalence of TTIs showed a significant increase from 2.44% to 3.71% (χ^2^ = 100.72, p = 0.00) over this 6-year period. The positive rates for TTIs varied along demographic lines. The top three risk factors in test-positive donors were identified as age, education level and donation frequency. The older age group and lower educated group were linked to a higher prevalence of TTIs. A decreasing prevalence was associated with an increasing frequency of blood donations (χ2 = 562.78, p = 0.00).

**Conclusions:**

Hepatitis B and C were found most, and often in conjunction with syphilis. These were the primary threats to blood safety. The high positivity rate and the increasing prevalence of TTIs among blood donors in Western China call for further actions.

## Introduction

Blood transfusion and component therapies are well-established and essential medical practices. These therapies, however, are not without risks and may lead to the transmissions of infectious agents from donor to recipient. Common infectious agents include hepatitis B virus (HBV), hepatitis C virus (HCV), human immunodeficiency virus (HIV) and syphilis–causing T. pallidum [1]. The HIV crisis related to blood donation in China has been well documented [2]–[4]. Collection of unhygienic plasma/blood in some rural areas of China during the mid-1990s led to regional HIV epidemics [5]. The Joint United Nations Program on HIV/AIDS (UNAIDS) reported that of 75,000 HIV infections in China in 2005, 22,000 were acquired through transfusions of contaminated blood [6]. In addition, many cases of HBV and HCV infections in Chinese adults were found to be associated with blood transfusions [2], [7]. HBV prevalence of 10% and HCV prevalence of 3.2% were observed in the general population meaning hepatitis was an endemic in China [8]. The prevalence of HCV infection among blood donors on the Chinese mainland from 1990 to 2010 was 8.68% [9].

In pursuit of global blood safety, the World Health Organization (WHO) recommends that all blood donations should be screened for evidence of infection prior to the release of blood and blood components for clinical or manufacturing use [10]. According to WHO guidelines, the screening of all blood donations should be mandatory for HIV, HBV, HCV and syphilis. Transfusion-transmissible infections (TTIs) have been drastically reduced in countries where routine serologic screening of donors is implemented [11], [12]. Nevertheless, there are still risks of TTIs due to the limitations of virus detection techniques. The median prevalence rate of TTIs in blood donations in middle- and low-income countries is much higher than that in high-income countries [13]. TTIs remain a significant threat to blood safety, causing grave concerns in many developing countries.

The prevalence of viral infections in blood donations can be used as a valuable indicator to assess the safety of blood supply and the potential risk of TTIs. Changes in this prevalence may also reflect trends in the infections of interests among the general population. At present, there is no comprehensive data about overall seroprevalence of major TTIs among blood donor groups in China, especially in underdeveloped western areas of China. In this study, we analyzed the data of screening tests on blood donations at a central blood center in Western China, examined the presence of hepatitis B virus surface antigen (HBsAg), anti-HCV, anti-HIV, and anti-TP (marker for syphilis infection) among blood donors, and explored the influencing factors of the detected results. Based on the findings, measures to improve blood safety through TTI prevention and control were suggested for policy-makers' consideration to minimize the risks of TTIs and safeguard public health in China.

## Methods

### Ethics Statement

The study protocol was approved by the Ethics Committee of the Institution of Chinese Medical Sciences, University of Macau. It did not require review in Western China since all the samples were anonymized. All donors at the central blood center in China were volunteers and, prior to blood donations, had to be verified for eligibility according to the current Chinese national blood donation criteria. Only those aged between 18 and 55 years, having a body weight above 50 kg for males and 45 kg for females, as well as physically and mentally fit would be accepted as eligible donors. In this study, written informed consent was obtained from all participants to allow the use of anonymized test results of their blood samples.

### Data collection

This cross-sectional study was performed in the largest multifunctional blood transfusion service center in Western China. Western China includes 10 provinces, municipalities and autonomous regions: Shaanxi, Gansu, Qinghai, Ningxia, Xinjiang, Sichuan, Chongqing, Yunnan, Guizhou, and Xizang. The region covers 5.38 million square kilometers, 56% of the land area of the country, and has a population of 287 million people, 22.99% of the national total [14]. In short, Western China is insufficiently industrialized and is relatively undeveloped compared with the eastern region of China. The sample site of the study is located in Sichuan Province, a large agricultural province and a key economic center of Western China, having a population of 80.42 million in 2010 [15]. The volume of donated blood averaged 40 tons each year at the sample site. Considering April was usually the donation peak month, data of all donated blood each April from 2005 to 2010 were requested and obtained for this study, amounting to 66,311 cases in total. The test results of these donations underwent anonymizations with the resultant information showing only donor demographic characteristics, donation frequencies and TTI screening results among the donors.

### Screening methods

At this central blood center, all potential donors first went through the pre-donation rapid testing procedures which tested for HBsAg. Then, all donor samples underwent two rounds of enzyme immunoassays (EIA) using two different reagents to test for anti-HIV-1, anti-HIV-2, anti-HCV and HBsAg. Syphilis antibody was also tested with Rapid Plasma Reagin (RPR) and enzyme-linked immunosorbent assay (ELISA) respectively. All reagents used in screening tests are listed in Table 1. All kits were approved and licensed by the Chinese State Food and Drug Administration. Blood samples were considered positive for TTI if any screening test showed positive results.

**Table 1:** Reagents and company used in donor screening.

Kit Name	Company
Diagnostic Kit for Antibody to HIV (ELISA)	Shanghai Kehua Bio-engineering Co., Ltd (KHB); bioMérieux Clinical Diagnostics
Diagnostic Kit for Antibody to HCV (ELISA)	Shanghai Kehua Bio-engineering Co., Ltd (KHB); Johnson & Johnson
Diagnostic Kit for Antibody to HBV Surface Antigen(ELISA)	Shanghai Kehua Bio-engineering Co., Ltd (KHB); InTec Products, INC (Xiamen)
Syphilis Rapid Plasma Reagin (RPR)	Shanghai Kehua Bio-engineering Co., Ltd (KHB)
Diagnostic Kit for Antibody to Treponema Pallidum (ELISA)	Beijing BGI-GBI Biotech Co., Ltd.

### Statistical analysis

Data were coded, entered, cleaned, validated and analyzed using IBM SPSS Statistics 19.0 (Armonk, New York, USA). The prevalence of TTIs was determined by the number of donations with positive TTI serologic markers in a year divided by the total number of blood donations in that year. The positivity rates of HBsAg, anti-HCV, anti-HIV, and anti-TP were expressed in percentages for the entire study group and groups with different demographic characteristics and donation frequencies. The comparisons between/among groups were performed using Fisher's exact test. Chi-square trend test was applied to examine the variation in trends. Logistic regression was employed to explore the influencing factors of the detected results: the dependent variable was the screening result (0 for negative and 1 for positive); the independent variables included gender, age, ethnic group, occupation, education level, donation frequency and the amount of donated blood.

## Results

### Study population

The characteristics of the study population are shown in Table 2. 51.07% were female and 48.93% were male. Their ages (in years) ranged from 18 to 55, with 18–29 years as the largest age group contributing to 43,401 donations (65.45% of donors). Among the donors, 97.8% were Han Chinese, 68.39% were single and 56.32% had a college degree or higher level of education. Nearly two-thirds were first-time donors and the percentage of first-time donation increased annually (χ^2^ = 637.57, p = 0.00).

**Table 2:** Demographic characteristics of blood donors (n = 66,311).

Variable		No. of donors	%
Gender	Female	33863	51.07
	Male	32448	48.93
Age	18∼	23683	35.72
	25∼	19718	29.74
	30∼	7083	10.68
	35∼	6638	10.01
	40∼	4867	7.34
	45∼	3076	4.64
	50∼55	1246	1.88
Ethnic group	Han Chinese	64855	97.80
	Ethnic minorities	1456	2.20
Marital status	Single	45350	68.39
	Married	20902	31.52
	Other	59	0.09
Education level	University education	37348	56.32
	Secondary education	27920	42.11
	Primary education	1043	1.57
Donor category	First time donor	39708	59.88
	Repeat donor	26603	40.12

### Major TTIs

Among the 66,311 blood donors, 1,769 were confirmed positive for at least one pathogen, giving a prevalence of TTIs at 2.67% (95% CI 2.55–2.79%). The overall positivity rates of HBsAg, anti-HCV, anti-HIV, and anti-TP were 0.87% (95% CI 0.80–0.94%), 0.86% (95% CI 0.79–0.93%), 0.31% (95% CI 0.26–0.35%), and 0.70% (95% CI 0.64–0.76%) respectively. 1,725 (2.60%, 95% CI 2.48–2.72%) of the donated blood had serological evidence of infection with only one pathogen and 44 (0.07%, 95% CI 0.05–0.09%) had multiple infections (Maximum = 3). The top three concurrent infections were HIV and syphilis (15), HBV and syphilis (14) and HCV and syphilis (8). Over this 6-year period, trend analysis for risks of TTIs showed a significant increase from 2.44% to 3.71% (χ^2^ = 100.72, p = 0.00). The results of confirmed viral markers tested in each year are presented in Table 3. Significant increases were noted in the prevalence of HCV, HIV and syphilis infections after 2007. The prevalence of HBV varied in a range between 0.70% and 1.07% from 2005 to 2010 with no statistical significance (p = 0.11). Overall, HBsAg was the most prevalent pathogen in the six years.

**Table 3:** Positivity rate of transfusion-transmissible infections, 2005–2010.

Year	N of donors	Positive(%)	HBsAg+(%)	Anti-HCV+(%)	Anti-TP+(%)	Anti-HIV+(%)
2005	6667	163(2.44)	54(0.81)	43(0.64)	49(0.73)	20(0.30)
2006	8602	166(1.93)	60(0.70)	41(0.48)	46(0.53)	20(0.23)
2007	8469	199(2.35)	76(0.90)	45(0.53)	68(0.80)	15(0.18)
2008	11509	253(2.20)	123(1.07)	46(0.40)	66(0.57)	24(0.21)
2009	13701	344(2.51)	112(0.82)	105(0.77)	90(0.66)	48(0.35)
2010	17363	644(3.71)	153(0.88)	289(1.66)	145(0.84)	76(0.44)
*P*-value		0.00	0.11	0.00	0.03	0.00
Total	66311	1769(2.67)	578(0.87)	569(0.86)	464(0.70)	203(0.31)

Note: *P*-value, Chi-square trend test to examine the variation in trends.

### Positivity rate by demographic characteristics

Table 4 shows the TTI positivity rates for groups with different demographic characteristics. The prevalence of TTIs was 2.66% for male and 2.67% for female in the donation population, without significant gender difference (p>0.05). HBV and syphilis infections were more prevalent among the males whereas HCV and HIV infections were more prevalent among the females, but none of the differences were significant (p>0.05). The prevalence of HBV, HCV, HIV and syphilis infections increased with age. The rate of TTI positive donations was higher in the married group. The prevalence of HBV, HCV, HIV and syphilis infections among married donors was 1.05%, 0.94%, 0.35% and 1.12%, respectively. The TTIs prevalence also showed significant differences among different education levels (χ^2^ = 72.47, p = 0.00). The positivity rate was lower in higher educated groups. Regarding occupation, farmers showed the highest incidence (4.30%) of TTIs while health care workers (2.35%) and students (2.27%) ranked as the bottom two.

**Table 4:** Positivity rate of transfusion-transmissible infections, by demographic characteristics, n(%).

	Donations	Positive(%)	HBsAg+(%)		Anti-HCV+(%)	Anti-TP+(%)	Anti-HIV+(%)
Gender							
Male	32448	864(2.66)	292 (0.90)		273 (0.84)	234 (0.72)	89 (0.27)
Female	33863	905 (2.67)	286 (0.84)		296 (0.87)	230 (0.68)	114 (0.34)
*P*-value		0.94	0.45		0.67	0.55	0.16
Age							
18∼	43401	1002 (2.31)	346	(0.80)	364 (0.84)	192 (0.44)	123(0.28)
30∼	13721	409 (2.98)	125	(0.91)	109 (0.79)	142 (1.03)	45 (0.33)
40∼55	9189	358 (3.90)	107	(1.16)	96 (1.04)	130 (1.41)	35 (0.38)
*P*-value		0.00	0.00		0.10	0.00	0.27
Marital status							
Single	45350	1062(2.34)	359 (0.79)		373 (0.82)	228 (0.50)	128 (0.28)
Married	20902	705 (3.37)	219 (1.05)		196 (0.94)	235 (1.12)	74 (0.35)
Other	59	2(3.39)	0 (0.00)		0 (0.00)	1 (1.69)	1 (1.69)
*P*-value		0.00	0.00		0.28	0.00	0.05
Occupation							
Student	25997	590 (2.27)	208 (0.80)		244 (0.94)	72 (0.28)	80 (0.31)
Technology worker	3333	91 (2.73)	35 (1.05)		27 (0.81)	26 (0.78)	5 (0.15)
Farmers	4953	213 (4.30)	63 (1.27)		61 (1.23)	72 (1.45)	24 (0.48)
Businessman	12940	333 (2.57)	109 (0.84)		94 (0.73)	98 (0.76)	37 (0.29)
Health worker	1236	29(2.35)	11 (0.89)		12 (0.97)	4 (0.32)	3 (0.24)
Public officials	2157	71 (3.29)	21 (0.97)		18 (0.83)	24 (1.11)	11 (0.51)
Others	15695	442 (2.82)	131 (0.83)		113 (0.72)	168 (1.07)	43 (0.27)
*P*-value		0.00	0.05		0.01	0.00	0.07
Education level							
Primary	1043	55 (5.27)	16 (1.53)		19 (1.82)	16 (1.53)	4 (0.38)
Secondary	27920	877 (3.14)	283 (1.01)		232 (0.83)	290 (1.04)	96 (0.34)
University or above	37348	837 (2.24)	279 (0.75)		318 (0.85)	158 (0.42)	103 (0.28)
*P*-value		0.00	0.00		0.00	0.00	0.27
Total	66311	1769(2.67)	578 (0.87)		569 (0.86)	464 (0.70)	203 (0.31)

Note:*P*-value, Fisher's exact test to test statistical difference in the distribution within each group

### Positivity rate by donation frequency

Among all donors, the prevalence of TTIs was 3.79% (95% CI 3.60–3.97%) for first-time donors, 1.29% (95% CI 1.09–1.48%) for donors who donated twice, and 0.71% (95% CI 0.57–0.86%) for donors who had donated three times or more (Table 5). A significant decrease in the prevalence was linked to the increased frequency of blood donations (χ^2^ = 562.78, p = 0.00). The most common TTI in first-time donors was HBV infection, followed by HCV infection.

**Table 5:** Positivity rate of transfusion-transmissible infections, by donation frequency, n(%).

	Donations	Positive(%)	HBsAg+(%)	Anti-HCV+(%)	Anti-TP+(%)	Anti-HIV+(%)
First time	39708	1503(3.79)	503(1.27)	477(1.20)	392(0.99)	169(0.43)
Twice	13305	171(1.29)	52(0.39)	56(0.42)	42(0.32)	24(0.18)
Three or more times	13298	95(0.71)	23(0.17)	36(0.27)	30(0.23)	10(0.08)
*P*-value		0.00	0.00	0.00	0.00	0.00
Total	66311	1769(2.67)	578(0.87)	569(0.86)	464(0.70)	203(0.31)

Note: *P*-value, Fisher's exact test to test statistical difference in the distribution within each group

### Influencing factors of TTI prevalence

In multivariable logistic regression analysis, the covariate variables were run as factor variables and the occurrence of TTI positive donations was independently associated with age, education level and donation frequency (Table 6). The regression result implied that: 1) more positive donations were found in the older age group; 2) lower educated donors were more likely to be confirmed positive for TTIs; 3) the TTI positivity rate was lower in repeat donors.

**Table 6:** Influencing factors of transfusion-transmissible infections prevalence, 2005-2010.

Variable	*P*-value	OR	95% CI
Age	0.00	1.03	1.02–1.04
Education level	0.00	0.75	0.68–0.82
Donation frequency	0.00	0.52	0.49–0.56

Note: OR, odd ratio; CI, confidential interval.

## Discussion

This study showed that 2.67% of the donated blood was seropositive for at least one of the screened markers. This number is comparatively low in China, as reports showed the seropositivity rate in donors ranged from 2.5% to 7% in other parts of China [16]. However, the seropositivity rate of 2.67% is considered very high compared with other developing countries [17], [18], posing great concerns about blood safety and promoting the need for stringent donor selection criteria and blood screening measures. It was also noted that 44 (0.07%) of donated blood specimens had serological evidence of multiple infections with syphilis and HIV as the most common concurrent infections, followed by syphilis and HBV. These coinfections should always be screened as a matter of priority.

In this study, the prevalence rate of HIV infection in the blood center in Western China (0.31%) was much higher than that in the general population (0.05%) [6]. This observation is not consistent across different areas of China. Given varying HIV prevalence rates among different regions in China, with Liaoning (0.22%) and Yancheng (0.18%) displaying a HIV prevalence rate among blood donors four to ten times higher than that in Guangzhou (0.02%) and Nanjing (0.08%) [19], further investigation is needed to explain this finding.

HBV has been a well-recognized public health concern in China for a long time. In compliance with international recommendations of vaccination to prevent and control HBV, a nationwide HBV vaccination program was implemented in 1992, and free HBV vaccination was provided for all neonates after 2005. Since then the prevalence of HBV infection in the general population has dropped significantly [20], [21]. With an effective vaccination coverage, the HBsAg carrier rate in the general population decreased from 9.8% to 7.2% in 2006. In particular, the prevalence of HBV infection decreased from 9–12% to 2.3% among children aged 5–14 years and was as low as 1.0% among children younger than 5 years in 2006 [21], [22]. Although the positive impact of the vaccination program on donated blood safety is too preliminary to establish at this stage, vaccination is considered as a highly effective measure to minimize HBV in donated blood in the long term. Meanwhile, the prevalence of HBV infection in China remains very high with approximately 100 million individuals having chronic HBV infection [23]. The significantly lower seroprevalence of HBV infection among the donor population (0.87%) in this study suggested that the pre-donation HBsAg rapid testing was critical for controlling risks.

The prevalence rate for HCV infection in blood donors was revealed to be 0.86% which is lower than that in the general population [8]. The Blood Donation Law implemented in 1998 in China, which only allowed voluntary blood donors, has played a major role in controlling the spread of HCV. In addition, the detection techniques for anti-HCV have been greatly improved, with better sensitivity and specificity in recent years. All these measures lead to a drastic decline in HCV prevalence throughout China after 1998. But infections of HBV and HCV remained common in this region.

Before 1998, TTIs were very common in China due to contaminated blood collections, particularly in for-profit plasma collection facilities. As the Blood Donation Law came into effect in 1998, blood collection was successfully shifted from paid and employer-organized donations to fully voluntary donations. The government also implemented a standardized national donor screening policy. The safety of blood supply and transfusion in China has been greatly enhanced. However, all the rates of TTI positive donations observed in our study showed an increased trend in the last two years, which is different from the decreasing trends reported in Western countries or other Asian countries [24]–[26]. This increase might be due to two reasons. Firstly, the sensitivity of reagents or methods used in blood screening tests has improved, leading to the increase in the number of positive confirmations. Secondly, the increasing trend of TTIs in the donated blood may be associated with the increased number of first-time donors in the last two years of the study. It has been well-documented that first-time blood donors always pose a greater risk of infectious donation than repeat donors [27]–[29]. Furthermore, the boost in the number of positive cases might be caused by people with suspected infections who opted for free blood screening tests at the center.

In accordance with other research results in China, our findings indicated that the TTI positive donations were independently associated with age, education level, and donation frequency [30], [31]. In terms of age, most of the younger donors were studying at university and were under supervision not only educationally but also socially. Almost all university students in China are required to live in boarding facilities bound by the strict regulations of the universities which might have compromised their social freedom. The younger generation would only have a relatively more active sex life, and thus higher disease susceptibility, upon graduation. As a result, the chance of infections was found to be low in the younger age group. More positive results of TTIs were also found in the low education level group. This could be explained by the fact that the higher educated groups have better understandings of screening educational materials and may defer from donations when at risk. This finding also conformed to the implication of Knowledge-Attitude- Practice (KAP) theory. In order to ensure long-term progress in blood supply safety, young people and higher educated groups should be encouraged to donate blood.

Furthermore, in line with other research, higher rates of TTIs were observed among first-time donors in our study and the prevalence decreased as the number of previous donations increased. Repeat donors might be better informed candidates who would deny risk behaviors before donations, and were tested negative at their prior donation so as to reduce the probability of blood infections in the window period. Moreover, it is believed that most repeat donors would not suddenly engage in high-risk behaviors. In order to help ensure the safety of blood supply, not only should new donors be carefully recruited to maintain the volume of the donation pool, but a cadre of previous donors should also be actively retained.

In conclusion, the seroprevalence of certain TTIs appeared to be lower among blood donors than that in the general population of China. This is largely due to the effective system to select donors and screen donated blood. However, hepatitis B and C were found most, and often in conjunction with syphilis. These were the primary threats to blood safety. The increasing trend of blood-borne infections in blood donors since 2007 calls for more stringent measures. More sensitive screening methods such as nucleic acid testing should be adopted to help detect TTIs earlier and thus reduce the risks associated with window periods. It is also considered highly important to improve donor recruitment procedures and increase the proportion of regular donors. Young people, higher educated groups and previous donors should be encouraged to donate blood to help ensure a long-term safe blood supply.
