# Correction: Prevalence and Trend of Major Transfusion-Transmissible Infections among Blood Donors in Western China, 2005 through 2010

**DOI:** 10.1371/journal.pone.0118426

**Published:** 2015-02-19

**Authors:** The PLOS ONE Staff

The following information is missing from the Funding section: This study was supported by the Research Fund of the University of Macau, MYRG106(Y1-L3)-ICMS13-BY. The funders had no role in study design, data collection and analysis, decision to publish, or preparation of the manuscript.
